# Catenary nanostructures as compact Bessel beam generators

**DOI:** 10.1038/srep20524

**Published:** 2016-02-04

**Authors:** Xiong Li, Mingbo Pu, Zeyu Zhao, Xiaoliang Ma, Jinjin Jin, Yanqin Wang, Ping Gao, Xiangang Luo

**Affiliations:** 1 State Key Laboratory of Optical Technologies on Nano-Fabrication and Micro-Engineering, Institute of Optics and Electronics, Chinese Academy of Science, Chengdu 610209, China

## Abstract

Non-diffracting Bessel beams, including zero-order and high-order Bessel Beams which carry orbital angular momentum (OAM), enable a variety of important applications in optical micromanipulation, sub-diffraction imaging, high speed photonics/quantum communication, etc. The commonly used ways to create Bessel beams, including an axicon or a digital hologram written to a spatial light modulator (SLM), have great challenges to operate at the nanoscale. Here we theoretically design and experimentally demonstrate one kind of planar Bessel beam generators based on metasurfaces with analytical structures perforated in ultra-thin metallic screens. Continuous phase modulation between 0 to 2π is realized with a single element. In addition, due to the dispersionless phase shift stemming from spin-orbit interaction, the proposed device can work in a wide wavelength range. The results may find applications in future optical communication, nanofabrication and super-resolution imaging, etc.

Diffraction is one of the intrinsic and fundamental properties of all types of waves, say, light, sound and matter waves etc. Since the discovery of the diffraction limit in the late 19^th^ century, diffraction of light has impeded the precise control of the light fields at sub-wavelength scale and became one of the major barriers faced by the optical community. In the last few years, several groundbreaking works such as extraordinary light transmission through subwavelength hole arrays^1^, beaming effect in groove-assisted slits^2^ and optical super oscillation effect^3^,^4^ have been reported by various groups, opening a door for the engineering of light-matter interaction for applications such as subwavelength light routing, colour filtering, super-resolution imaging and quantum signal processing^1^,^2^,^3^,^4^,^5^,^6^.

Practically, all kinds of light waves are suffering from the diffraction-induced shape change during the propagation in free-space, and it is well known that the beam width of a Gaussian shaped laser beam changes dramatically especially if the initial beam width is not much larger than the operating wavelength. In 1987, Durnin *et al.*, showed that there exists one kind of non-diffracting beam which has Bessel-type intensity distribution along the radial direction^7^. This so-called Bessel beam is also an exact solution of the Helmholtz equation, thus does not violate the traditional optical theory. However, the ideal Bessel beam requires the horizontal dimensions as well as the power carried by the beam is infinite, which cannot be achieved in practical situations. Nevertheless, it was shown that even truncated with a Gaussian function, the Bessel beam can still maintain its unique properties such as diffraction-free and self-healing, which enable a variety of important applications in super-resolution imaging^8^, optical micromanipulation and nanofabrication^9^,^10^.

Very recently, high-order Bessel beams (HOBBs), i.e., Bessel beams carrying orbital angular momentum (OAM) have attracted growing interest owing to their ability to transport information encoded in the OAM basis^11^,^12^. The diffraction-free property of the Bessel beam, combined with the (theoretically) infinite freedoms of OAM, makes the HOBBs become a promising alternative for high-speed optical and quantum communications systems. Besides, the particular shape of the HOBBs and its ability to retain over an extended propagation distance in a propagation-invariant manner makes it useful in optical manipulation^10^.

Zero-order Bessel beams can be generated by using either refractive optical elements (axicons) or diffractive optical elements such as computer generated holograms (CGHs)^13^. Similarly, HOBBs can be obtained by illuminating an axicon with a Laguerre-Gauss (LG) beams or propagating a zeroth-order beam through a spiral phase plate. In recent years, more compact devices such as a spatial light modulator (SLM) were used to create HOBBs. However, the pixel size of a typical SLM is at least 6.4 × 6.4 μm, which is more than one order of magnitude larger than the wavelength of visible light, which limited the performance of such devices. More recently, metasurfaces, the equivalent two-dimensional (2D) metamaterials have been used in the wavefront engineering in free space. With these additional phases generated by the meta-atoms, the wavefront could be shaped on demand to construct many novel optical devices, such as beam deflectors^14^,^15^,^16^, focusing lenses^17^,^18^,^19^, spiral phase plates^14^,^20^, hologram^21^,^22^, to name a few. Furthermore, diffraction engineering on the structure’s surfaces was also realized, including beam engineering for polarization-controlled directional coupling^23^, Airy beams^24^, collimated beams^25^, and other arbitrary beams^26^,^27^. The discretely arranged optical antennas in the metasurfaces allow one to sample the phase or amplitude distribution in subwavelength scales, providing a substantial improvement of the performances. Nevertheless, most current metasurfaces are limited by the collective response of meta-atoms, which means that a single meta-atom/molecule is not competent for the phase manipulation. Furthermore, the discrete phase sampling in traditional metasurfaces would introduce significant noise to the generated optical fields.

In this article, we propose and demonstrate a design, which combines ultrathin planar micrometer/sub-micrometer grating and analytically designed nano-slits, to create Bessel beams. The quasi-continuous phase modulation of our proposed nano-slit structures makes its pixel to be extremely small. Furthermore, these generators can also operate in a wide frequency range, since the geometric phase is intrinsically independent of the operational wavelength.

## Results

### Principle and design of Bessel beam generators

**Figure 1:**
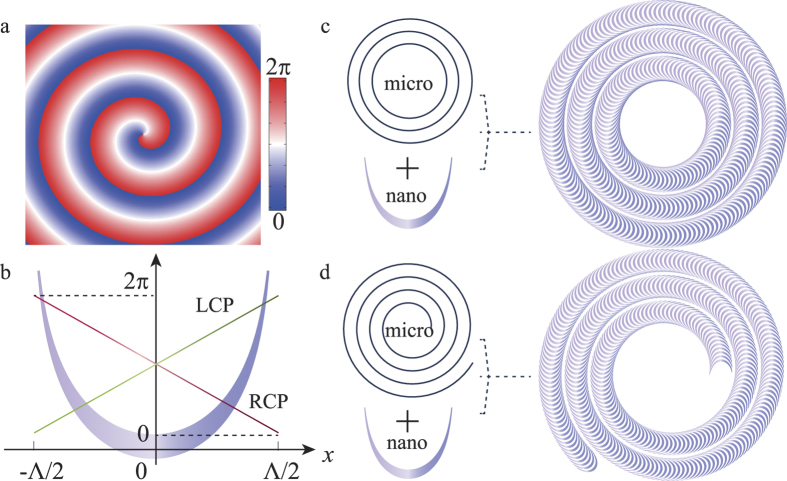
Schematic of the Bessel beam generation process in catenary arrays. (**a**) Phase pattern of a Bessel beam with *l* = 1. (**b**) Horizontal phase distributions in [0, 2π] under illumination of circular polarizations for a catenary aperture. (**c,d**) Sketch of the Bessel beam generators for *l* = 2 and 3.

A general design procedure for Bessel beams generators can be obtained from the principle of holography. The electromagnetic fields at any cross section perpendicular to the propagation direction can be written as:


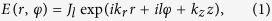


where *J*_*l*_ is the *l*^th^-order Bessel function, *k*_*r*_ is the wave vector along the radial direction, *φ* is the azimuthal angle, and *l* is the topological charge of the optical vortex. The complex amplitude of such a Bessel beam can be recorded and then used as a holographic Bessel beam generator. As shown in Fig. 1a, the phase distribution at *z* = 0 is calculated according to equation (1), where *k*_*r*_ = 0.15*k*_0_, and *k*_0_ = 2π/λ.

To obtain the corresponding phase profile with metasurface, we utilize the concept of phase discontinuity at the metasurface. In contrast to the previous metasurface comprised of discrete metallic^28^ or dielectric scatters^18^,^29^, our structures were designed to be continuously changing along the coordinates and obtain quasi-continuous phase distribution based on the analytically designed equation^30^. As illustrated in Fig. 1b–d, our design can be treated as a combination of nano-slit with a micrometer/sub-micrometer scale grating, which is concentric rings or spirals, acting as a zone plate or spiral zone plate in traditional optics. The shape of the nano-slit can be described by the natural catenary curve^31^,^32^


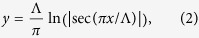


where Λ is the horizontal length of the curve and the range of *x* for a single unit cell is within (-Λ/2, Λ/2). In the following, we term this kind of structures as catenaries^30^,^31^,^32^. Under circularly polarized illumination, the transmitted complex light fields can be further decomposed into two different handed circularly polarized components, whereas the cross-polarized component acquires a gradient phase:^16^,^33^,^34^


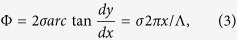


where *σ* = ±1 denotes the right-circularly polarized (RCP) light beam and left-circularly polarized (LCP) light beam, *arc*tan(*dy*/*dx*) is the angle between the tangent line to the catenary curve and the *x*-axis. Since the circular polarization and phase gradient are respectively related to the spin and linear momentum of photon, equation (3) is actually a manifestation of the spin-orbit interaction.

For the simplicity of discussion, we only consider the phase distribution of σ = 1 (For σ = -1, the phase profile will be reversed in sign). The single catenary can be arranged in the polar coordinates to achieve phase retardations along both the radial and azimuthal directions. Obviously, the phase difference between the two ends of the catenary is ± 2π, thus the trajectory of the catenary ends form the concentric or spiral gratings, which can be written as:


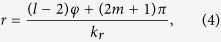


where *m* = 1, 2, 3, …. Figure 1c,d depict the two designs for *l* = 2, and 3, respectively. It is worth noting that our approach of design can surpass the restriction of continuous subwavelength gratings structures for forming a desired complex phase function^34^,^35^.

### Experimental characterization of Bessel beam generators

**Figure 2:**
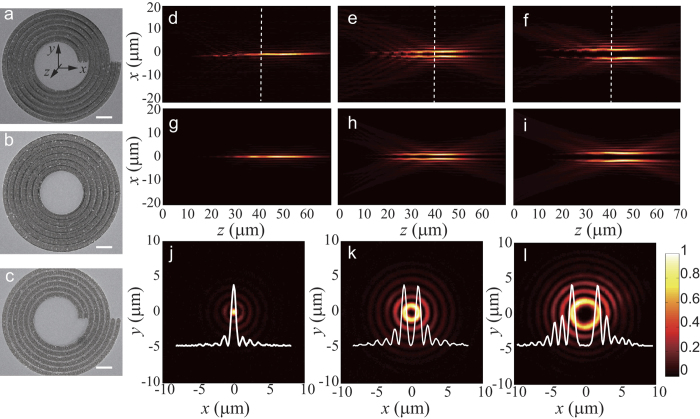
Optical characterization of the Bessel beams generators. Scanning electron microscopy images of generators for *l* = 0 (**a**), *l* = 2 (**b**) and *l* = 4 (**c**). Scale bar, 5 μm. (**d**–**f**) Experimental results of the intensity distribution in the *xz* plane (*y* = 0) for *l* = 0, 2 and 4. (**g**–**i**)Calculation results of the intensity distribution in the *xz* plane (*y* = 0) for *l* = 2, 3 and 4. (**j**–**l**) Cross-section view of the intensity along the dashed line marked in (**d**–**f**). Insets show the normalized intensity at *y* = 0.

Following the analytic design, Bessel beam generators for topological charges *l* = 0, 2, 3 and 4, were fabricated in our experiment. All the samples are milled in an Au film on a quartz substrate using a focused ion beam (FIB, FEI Helios Nanolab 650). The thickness of the Au film is 120 nm. Scanning electron microscopy (SEM) images of *l* = 0, 2, and 4 are shown in Fig. 2a–c. The period *p* in the radial direction is 2 μm for all the samples, which determines the radial wave vector of the diffraction beam, by the relation *k*_*r*_* = *2π*/p*.

In our experiment, measurements of the diffraction-free transmission properties were performed by using a home-built microscope^20^. A collimated beam from a He-Ne laser at λ = 632.8 nm was converted into right-hand polarization (RCP) light through cascaded polarizer and quarter-wave plate, and then illuminated on the samples. The cross-polarization (LCP) component of the transmitted field through the samples, was filtered by an additional quarter-wave plate and polarizer. The intensity distribution of the fields was imaged through a 100 × objective and a tube lens, then collected by a charge-coupled device (CCD, 1600 × 1200 pixels, WinCamD-UCD15, DataRayInc) camera. We measured the intensity distribution of the fields in a serial image planes by moving the objective along the *z* direction (propagation direction of the Bessel beam) with a step of 1 µm, then mapped the longitudinal cross-sections of the generated Bessel beams. The output surface of the sample was set as the *z* = 0 plane, and the center of beam spot in each *xy* plane was set as the original for *x* and *y* coordinate. Figure 2d–f show the experimental results of the intensity distributions across the centre of Bessel beams for *l* = 0, 2 and 4 in the *xz* plane (*y* = 0). The theoretical calculation based on vectorial angular spectrum theory^36^ is also given as illustrated in Fig. 2g–i, and good agreement with experimental data is found. The propagation-invariant manner of the Bessel beams was validated in both experimental and theoretical simulations. In particular, the HOBBs are characterized by hollow centre, a typical property of beams carrying OAM. As depicted in Fig. 2j–l, the spot size, defined as the radius of the innermost ring of the Bessel beam, increases with the topography charge *l* for the same radial wavevector and wavelength. Meanwhile, the spot size can also be manipulated by changing the period *p* of the microscale/sub-microscale grating. For reduced *p*, the radius of spot of Bessel beam can be even subwavelength, which is not realizable by the traditional SLM devices, due to the large pixel size.

**Figure 3:**
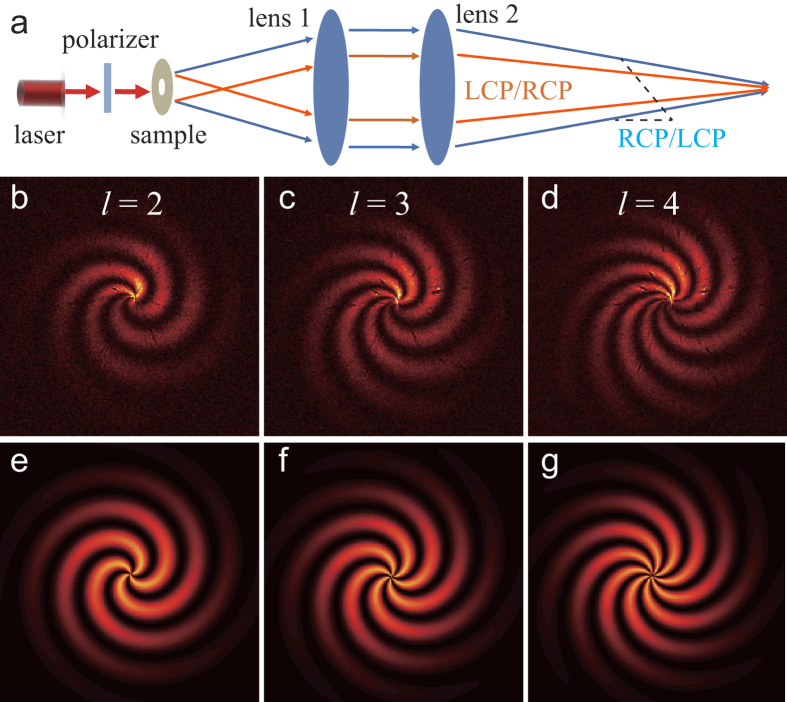
Interference characterization of the OAMs of HOBBs. (**a**) Experimental Setup. (**b–d**) Experimental interference patterns for *l* = 2, 3 and 4. (**e–g**) Calculated interference patterns for *l* = 2, 3 and 4.

Interferometry, a commonly used method to characterize the specific type of beam carrying OAM, was adopted to characterize the topography charge of the HOBBs. The schematic of the characterization experiment is shown in Fig. 3a. We used a linear polarized (*x*-polarized) light to irradiate the samples. After spin-orbital interaction in the samples, the converted RCP and LCP components of the beam were diverged at an angle of 2*θ*, where *θ* equals to sin^−1^(λ/*p*). After diverging, the RCP and LCP beams carry OAM with ± *l* respectively. The two beams were then collimated and focused by two lenses with relatively large area. The *y*-polarized components of the interference patterns around the focus point were recorded by a large area CCD camera (USB L11059, Ophir Inc.) with 35 × 24 mm active area. The interference patterns of *l* = 2, 3 and 4 from experiment are illustrated in Fig. 3b–d, which elegantly agree with the calculation results as shown in Fig. 3e–g. The petal-like intensity patterns originate from the interference between the beam carrying OAM with opposite signs. The number of the petals in the intensity patterns is the twice of |*l|*.

**Figure 4:**
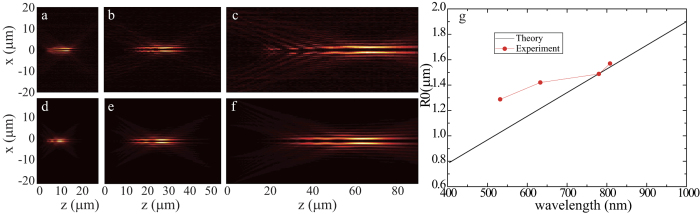
Broadband response of the Bessel beam generators. (**a–c**) Experimental intensity distributions in the *xz* plane at wavelengths of λ = 808 nm (**a**), 780 nm (**b**) and 532 nm (**c**) for *l* = 3. (**d–e**) The corresponding theoretical results at λ = 808 nm, 780 nm and 532 nm. (**g**) The radii of the Bessel beams for various wavelengths with *l* = 3.

As the phase distribution is determined by the geometric design, the proposed structure inherently works in a wide frequency range, due to the dispersion-less phase distribution. We measured the propagation property of light with *l* = 3 at a variety of wavelengths in our experiment. Figure 4a–c show the experimental results of the intensity distributions across the centre of beams at wavelength λ = 808 nm, 780 nm and 532 nm. Theoretically, the bandwidth of our proposed structures is not limited unless the wavelength is too short that *k*_r_ is larger than the wave vector in free space. The theoretical propagation properties of the HOBBs at the corresponding wavelengths were also calculated, as shown in Fig. 4d–f. The results from the theoretical model and experimental measurement match well. Figure 4g illustrates the wavelength dependent spot size with OAM *l* = 3. The spot size of the HOBBs is linearly dependent on the operating wavelength approximately. The small discrepancy between the experimental data and the theoretical model is owing to the fabrication and measurement errors.

## Conclusion

In summary, we proposed a novel catenary-shaped nanostructure to generate Bessel beams carrying various angular momenta. Different from traditional metasurfaces, the catenary structure could generate semi-continuous geometric phase in 2D space. Owing to the greatly reduced effective pixel size, higher energy efficiency could be expected compared to the discrete scatter based metasurfaces. Furthermore, the phase modulating properties of the catenary structures are independent on the operational frequencies, which provide a way to construct broadband Bessel beam generator via an ultrathin layer. In addition, the catenary metasurface may also serve as a platform to enhance the circular-polarization selective effects such as circular dichroism and optical activity^37^.

## Additional Information

**How to cite this article**: Li, X. *et al.* Catenary nanostructures as compact Bessel beam generators. *Sci. Rep.* **6**, 20524; doi: 10.1038/srep20524 (2016).
